# Factors affecting the production and measurement of hydrogen peroxide in honey samples

**DOI:** 10.1099/acmi.0.000198

**Published:** 2021-01-28

**Authors:** Annabel Guttentag, Krishothman Krishnakumar, Nural Cokcetin, Elizabeth Harry, Dee Carter

**Affiliations:** ^1^​ School of Life and Environmental Sciences and the Marie Bashir Institute for Infectious Diseases and Biosecurity, University of Sydney, Sydney, NSW 2006, Australia; ^2^​ ithree Institute, University of Technology Sydney, Sydney, NSW 2007, Australia

**Keywords:** catalase, colour, colourimetric assay, honey, horseradish peroxidase, hydrogen peroxide, Pfund

## Abstract

Many Australian native honeys possess significant antimicrobial properties due to the production of hydrogen peroxide (H_2_O_2_) by glucose oxidase, an enzyme derived from the honeybee. The level of H_2_O_2_ produced in different honey samples is highly variable, and factors governing its production and stability are not well understood. In this study, highly active Australian honeys that had been stored for >10 years lost up to 54 % of their antibacterial activity, although almost all retained sufficient activity to be considered potentially therapeutically useful. We used a simple colourimetric assay to quantify H_2_O_2_ production. Although we found a significant correlation between H_2_O_2_ production and antibacterial activity across diverse honey samples, variation in H_2_O_2_ only explained 47 % of the variation observed in activity, limiting the assay as a screening tool and highlighting the complexity of the relationship between H_2_O_2_ and the killing power of honey. To further examine this, we tested whether H_2_O_2_ detection in honey was being inhibited by pigmented compounds and if H_2_O_2_ might be directly degraded in some honey samples. We found no correlation between H_2_O_2_ detection and honey colour. Some honey samples rapidly lost endogenous and spiked H_2_O_2_, suggesting that components in honey, such as catalase or antioxidant polyphenols, may degrade or quench H_2_O_2_. Despite this rapid loss of H_2_O_2_, these honeys had significant peroxide-based antibacterial activity, indicating a complex relationship between H_2_O_2_ and other honey components that may act synergistically to augment activity.

## Introduction

Honey is widely known as a supersaturated sugar solution, primarily consisting of fructose and glucose. However, it is also a complex mixture containing proteins of both plant and bee origin, as well as antioxidants, polyphenols, flavonoids and Maillard reaction products [1–3]. The broad-spectrum antimicrobial activity of honey against fungi and bacteria has been characterized extensively [4–6]. Many honeys exhibit high antimicrobial activity due to hydrogen peroxide (H_2_O_2_) production from bee-derived glucose oxidase, which becomes activated upon dilution with water [7].

Australia has a rich diversity of native flora, and many Australian native honeys have been found to have high H_2_O_2_-based antibacterial activity, particularly those derived from *Eucalyptus marginata* (jarrah) and *Corymbia calophylla* (marri) [8]. However, H_2_O_2_-based antimicrobial activity can be highly variable across samples, even when these are derived from the same botanical source [8–10]. Storage and processing conditions affect glucose oxidase, which is vulnerable to damage from heat and light [7, 10, 11]; bee factors such as colony health and diet may influence H_2_O_2_ production [12], and floral components including plant-derived catalases (introduced through pollen) and antioxidants can quench H_2_O_2_ [13, 14]. This variability, along with the difficulty of accurately determining H_2_O_2_ production, has limited medical interest and clinical uptake of peroxide-type honeys.

The current test for measuring antimicrobial activity in honey uses a standard well diffusion assay, where zones of inhibition produced by honey samples are measured against phenol standards [5, 15]. Diluted honey, with or without catalase, is tested against *Staphylococcus aureus* to determine hydrogen peroxide (PA) or ‘non-peroxide’ activity (NPA), respectively [5]. Based on the capacity to inhibit wound pathogens, an NPA or PA ≥10 is considered to be potentially therapeutically active [16, 17]. This method is still used by some commercial honey suppliers, but it is laborious and can be difficult to reproduce and analyse objectively [18]. Various chemical assays have been employed by research laboratories to quantify H_2_O_2_ production, but these are generally expensive or limited in sensitivity, restricting their use in high-throughput analyses [19].

Recently, our group published a low-cost protocol based on a colourimetric horseradish peroxidase (HRP) assay to measure H_2_O_2_ production in honeys [20]. However, the correlation between H_2_O_2_ detected by this assay and antibacterial activity determined through the well diffusion assay is weak [8, 10], with pigmented antioxidants and Maillard reaction products present in honey suggested to interfere with test output [20].

The aim of the current study was to examine a set of honey samples that had been found to have high H_2_O_2_-dependent antimicrobial activity and to test (a) whether this activity was stable over 11–12 years of storage and (b) how activity correlated with H_2_O_2_ assessed using the HRP test. We show that over time the honey samples experienced a decline in antimicrobial activity, although almost all retained enough activity to remain potentially therapeutically useful. Some honey samples rapidly lost endogenous or spiked H_2_O_2_ following dilution, suggesting that there are compounds present that directly degrade H_2_O_2_, but these do not necessarily eliminate antimicrobial activity. H_2_O_2_ production, as quantified chemically by the HRP assay, was not able to explain all of the variation observed in the antibacterial activity of the different honey samples, suggesting a complex relationship between H_2_O_2_ and antimicrobial action.

## Methods

### Honey samples

This study used 14 honey samples that had been collected during an Australian honey survey and had been found to have a potentially therapeutic level of antimicrobial activity (equivalent to ≥10 % phenol, which is the level required to kill most wound pathogens) (Table 1) [8, 16]. All samples had been stored in the dark at 4 °C since their collection and were retested to determine any change in activity and verify that they remained active. Barnes Naturals Active Jarrah Honey (Barnes 10+), a commercial honey with activity attributed to H_2_O_2_ production, and Jarrah 2017, a jarrah honey sample collected in 2017, were included as fresh and commercial honey samples, respectively (Table 1). Artificial honey was used as an osmotic control and was created by mixing 160 g fructose (41.6 %), 144.8 g glucose (37.3 %), 11.2 g sucrose (2.9 %) and 70 g deionized water (18.2 %) with shaking in a 40 °C water bath until homogenous in consistency.

**Table 1.** Honey samples used in this study, including antimicrobial activity before and after storage and their level of H_2_O_2_ production

Honey sample*	Floral source	Land type	Region, state	Original antimicrobial activity†	Retested antimicrobial activity‡	Difference in antimicrobial activity (%)	Maximum H_2_O_2_ production§
Banksia 11	*Banksia* sp.	State forest	Illawarra, NSW	17.1±0.9	10.3±0.8	−6.8 (−40 %)	1.14±0.11
Jarrah 2017	*E. marginata*	Woodland	Northcliffe, WA	na	12.9±0.9	na	1.59±0.18
Jarrah 5	*E. marginata*	Woodland	Lower West, WA	25.4±0.4	18.4±1.1	−7.0 (−28 %)	2.95±0.08
Jarrah 8	*E. marginata*	Woodland	Lower West, WA	25.1±0.9	17.1±0.4	−8.0 (−32 %)	2.77±0.18
Jarrah 10	*E. marginata*	Woodland	Lower West, WA	25.7±0.5	17.5±0.4	−8.2 (−32 %)	2.86±0.31
Jarrah 13	*E. marginata*	Woodland	Lower West, WA	28.1±0.1	23.4±1.1	−4.7 (−17 %)	3.84±0.24
Karri 3	*E. diversicolor*	Woodland	Southwest, WA	29.6±0.7	25.7±0.6	−3.9 (−13 %)	2.22±0.07
Marri 6	*E. calophylla*	Urban, nature reserve	Lower West, WA	28.6±0.9	24.0±0.8	−4.6 (−16 %)	1.44±0.07
Marri 8	*E. calophylla*	National Park	Lower West, WA	27.2±0.2	24.3±0.6	−2.9 (−11 %)	2.38±0.13
Marri 10	*E. calophylla*	Farm, agricultural – stock, woodland	Lower West, WA	29.3±1.2	20.7±0.8	−8.6 (−29 %)	2.06±0.01
Marri 11	*E. calophylla*	Farm, agricultural – stock, woodland	Lower West, WA	29.7±0.1	25.2±0.8	−4.5 (−15 %)	2.54±0.03
Polyfloral 12	Mixed	Urban	Metropolitan, NSW	19.4±0.1	9.8±0.7	−9.6 (−49 %)	0.21±0.01
Polyfloral 13	Mixed	Urban, nature reserve	Metropolitan, NSW	21.2±0.6	17.7±0.9	−3.5 (−17 %)	0.29±0.03
Stringybark 11	*Eucalyptus macrohynca*	Farm, state forest	North Central, VIC	23.3±1.1	11.1±0.9	−12.2 (−52 %)	0.24±0.06
Stringybark 19	*Eucalyptus* sp.	Woodland	Northern Tablelands, NSW	24.9±1.2	11.4±0.4	−13.5 (−54 %)	0.93±0.12
Barnes 10+ honey	*E. marginata*	Not known	Not known	na	9.6±0.9	na	0.08±0.01

*Obtained from [8], except for Jarrah 2017 and commercial Barnes 10+ honey.

†Assessed as % phenol equivalence in 2006/7; data are the mean±sem of two biological replicates [8].

‡Assessed as % phenol equivalence in 2018; data are the mean±sem of two biological replicates.

§Quantified in mM using the horseradish peroxidase colourimetric assay; data are the mean±sem of two biological replicates.

### Quantification of antibacterial activity by bioassay

The antibacterial activity of the honey samples was determined using the standardized well diffusion assay outlined by Allen *et al*. [5], with minor modifications as specified elsewhere [8]. In this assay, freshly diluted honey with or without the addition of catalase was tested against *S. aureus* (ATCC 25923) to determine PA or NPA, respectively [5]. The resulting zones of inhibition were compared to the zones of phenol standards to give antibacterial activity as a measure of % phenol equivalence (w/v).

### Spectrophotometric measurement of honey colour

Honey colour was determined using a previously described method [21, 22]. Briefly, ~1 g honey aliquots were placed in a 50 °C water bath to dissolve crystallized sugars, then diluted with an equal volume of dH_2_O and optical density was measured at 635 nm using a UV/Vis spectrophotometer (UV-1600 PC, VWR International, Pty Ltd) with dH_2_O used as a blank.

Pfund values (measured in mm) are used by the agricultural industry to define honey colour [23]. The Pfund honey grader is an instrument that visually compares liquid honey to an amber glass standard, where the distance along the glass standard (1–140 mm) describes the honey colour or Pfund value. Absorbance readings are converted to Pfund values using the equation described in [21]:

Pfund (mm)=−38.70+371.39(*A*_635_).

### Quantification of H_2_O_2_ production in honey

A recently described standardized protocol was used to quantify H_2_O_2_ production using the horseradish peroxidase (HRP)/*o*-dianisidine colourimetric assay [20]. In the presence of H_2_O_2_, horseradish peroxidase oxidises colourless *o*-dianisidine to a coloured product. In this protocol, 2 g honey aliquots were diluted to 50 % (w/v) in dH_2_O and incubated at 35 °C with shaking at 180 r.p.m. for 20 min to aid mixing. Solutions were filter-sterilized and further diluted to 25 % (w/v) in dH_2_O and were vortexed until frothy. The diluted honey samples were then placed in 28 ml McCartney bottles to allow for adequate aeration and incubated at 35 °C for up to 6 h.

H_2_O_2_ measurements were taken every hour to generate a time course. For each measurement, 40 µl of honey prepared as above, 135 µl of colourimetric reagent solution [50 µg ml^−1^ *o*-dianisidine (Sigma Aldrich, cat. no. D9143) and 20 µg ml^−1^ horseradish peroxidase (Sigma Aldrich, cat. no. 31490) in phosphate-buffered saline (PBS)] were added to a 96-well flat-bottomed microtitre plate and incubated at room temperature for 5 min. For honey blanks, 135 µl of PBS was added in place of the colourimetric reagent solution. The reaction was stopped by the addition of 120 µl of 6 M H_2_SO_4_, which forms a stable purple *o*-dianisidine product that was measured by absorbance at 550 nm in a plate reader (BioTek, Millennium Science). To generate a standard curve, H_2_O_2_ (Sigma Aldrich, cat. no. 88597) was serially diluted in PBS to give final concentrations ranging from 2.1 to 2000 µM. The linear portion of the H_2_O_2_ standard curve was used to quantify H_2_O_2_ production in the honey samples.

### Investigation of assay inhibition

To investigate whether inhibitory factors were present in honey samples and were interfering with the H_2_O_2_ assay, recovery of a spiked aliquot of H_2_O_2_ was determined for honey samples Barnes 10+ and stringybark 11, which are antimicrobially active but have little H_2_O_2_ production [20]. Honey samples were prepared as outlined above and following 2 h of incubation were spiked with H_2_O_2_ to give a final concentration of 500 µM. The level of H_2_O_2_ recovered by the assay was determined by comparison with an artificial honey control.

### Data analysis

All data were analysed using GraphPad Prism 7. The D’Agostino–Pearson omnibus normality test confirmed the Gaussian distribution of data sets. Pearson correlation analysis and linear regression analysis were used to describe the correlations and linear relationships, respectively, between the means of continuous variables, antibacterial activity, H_2_O_2_ production and the Pfund colour of honeys. The Pearson’s correlation coefficient (*r*) describes the magnitude and the direction of the correlation.

## Results

### Honey activity and stability over time

Honey samples from the survey by Irish *et al.*, 2011 [8] were initially tested for antimicrobial activity (% phenol equivalence) in 2006 and 2007. In the current study we retested their activity following 11–12 years in storage in the dark at 4 °C. Although the honey samples all declined in activity over this time (ranging from 11–54 %; Table 1), almost all retained activity to a level considered therapeutically beneficial [≥10 % (w/v) phenol], with only two (Banksia 11 and Polyfloral 12) dropping to borderline activity [16]. Jarrah and marri honey samples lost similar amounts of activity, ranging from 17–32 % and 11–29 %, respectively (Table 1), while polyfloral and stringybark honey samples showed greater declines, ranging from 17–49 % and 52–54 %, respectively (Table 1).

### Honey colour

**Fig. 1.**
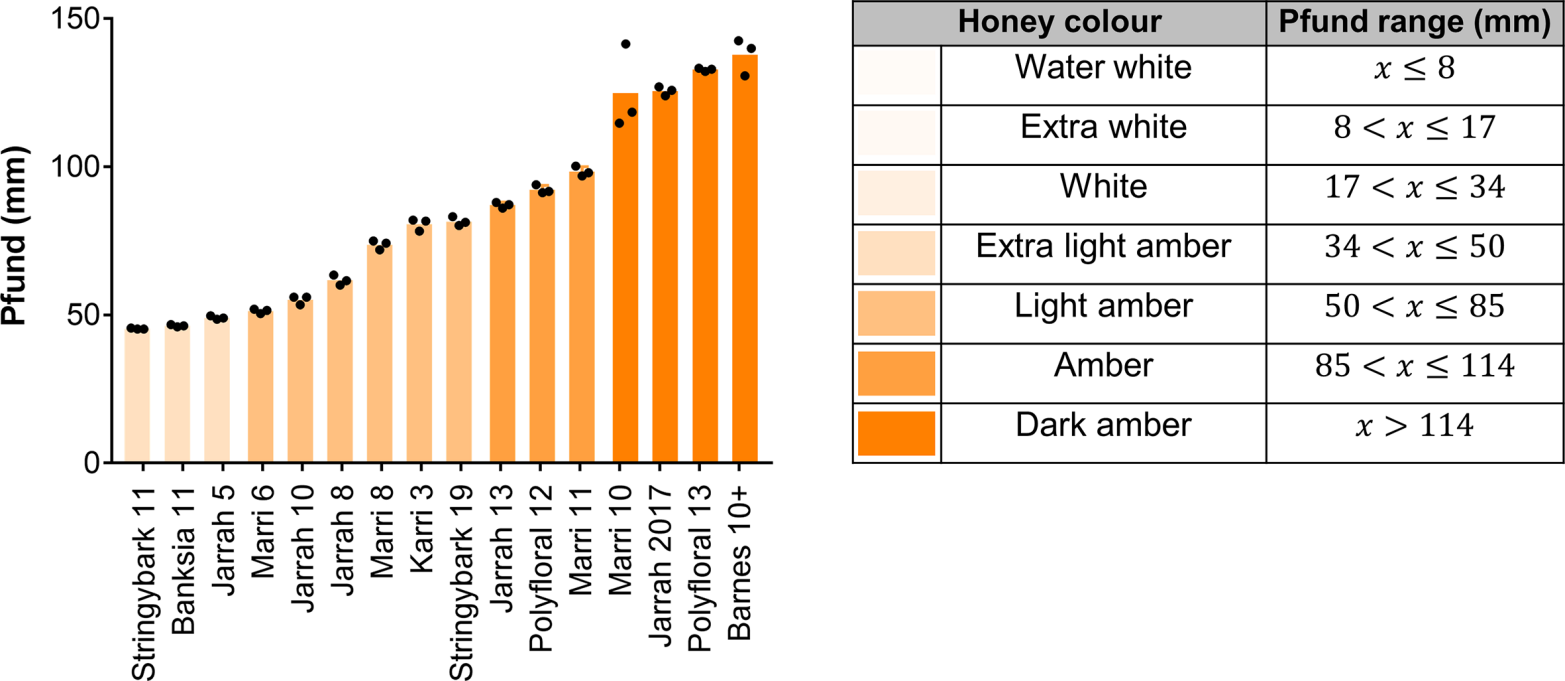
High H_2_O_2_ -producing honey samples were diverse in colour. Pfund (mm) values for the 16 honey samples. *n*=3 technical replicates; bars represent the mean.

Honey samples were organized into Pfund colour categories according to previous agricultural standards [21], which range from water white (Pfund ≤8) to dark amber (Pfund >114) (Fig. 1). The samples covered a spectrum of colour, ranging from extra light amber (34 <Pfund ≤50) to dark amber (Pfund >114) (Fig. 1) Different jarrah and marri samples varied substantially in colour despite originating from the same floral source.

### Correlations between honey H_2_O_2_, antimicrobial activity and colour

**Fig. 2.**
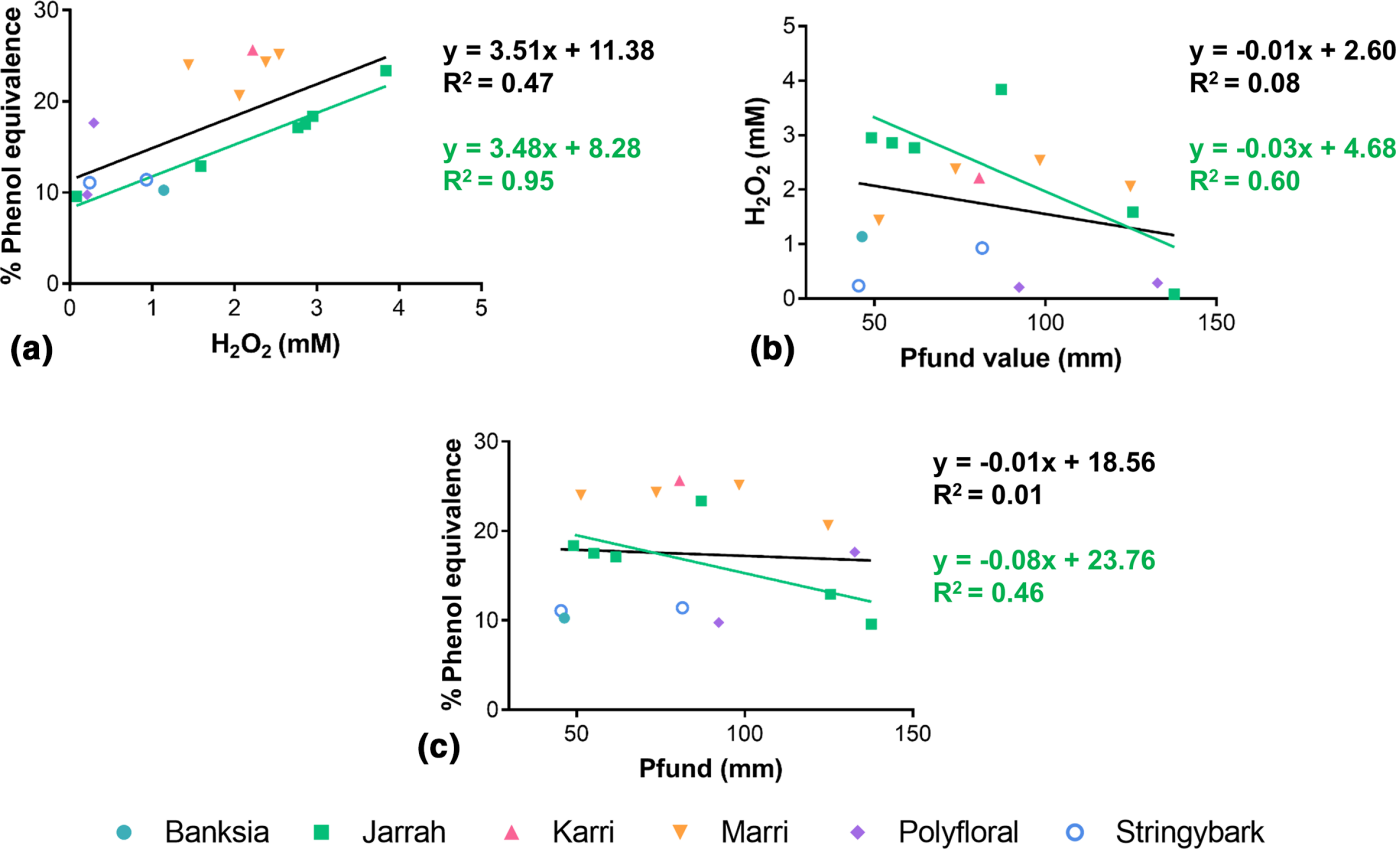
Correlations between antimicrobial activity (% phenol equivalence), H_2_O_2_ production and colour (Pfund). (**a**) A positive correlation was seen between H_2_O_2_ production and activity across honey samples (*r*=0.068, *P*=0.004; black line), but the explanatory power was low (*R* ^2^=0.47). The correlation was strengthened when restricted to jarrah samples (*r*=0.97, *R* ^2^=0.95, *P*=0.001; green line). (**b**). Honey colour and H_2_O_2_ production had a slight negative trend for all honey samples (black line) and jarrah samples (green line), but neither was significant (*P*=0.28 and *P*=0.07, respectively). (**c**) There was no correlation between honey colour and antimicrobial activity across all honey samples (black line) or jarrah samples (green line) (*P*=0.79 and 0.14, respectively). *n*=1 biological replicate (with three technical replicates) for Pfund values, and *n*=2 biological replicates (each with two technical replicates) for H_2_O_2_ readings and % phenol equivalence. The means of these data sets are shown in both graphs.

The antimicrobial activity of peroxide-type honeys (determined by the % phenol equivalence bioassay) should correlate directly with their level of H_2_O_2_ production, as all activity is abolished in the presence of catalase. Across all the honey samples tested, the maximum H_2_O_2_ production detected by the HRP assay had a positive correlation with antimicrobial activity (*r*=0.68, *P*=0.004) (Fig. 2a), but, the variation in H_2_O_2_ only explained 47 % of the variation in activity (*R* ^2^=0.47). Departure from a clear association was seen with polyfloral honey samples 12 and 13, which had similar H_2_O_2_ levels at 0.21±0.01 and 0.29±0.03, but substantially different activity levels at 9.8±0.7 and 17.7±0.9, respectively (Table 1, Fig. 2a). Similarly, marri samples 6 and 8 had divergent H_2_O_2_ levels at 1.44±0.07 and 2.38±0.13, but similar antimicrobial activity levels at 24.0±0.07 and 24.3±0.6, respectively (Table 1, Fig. 2a). When restricted to the seven jarrah honey samples, however, there was a very strong linear correlation between H_2_O_2_ and activity (*r*=0.97, *R* ^2^=0.95, *P*=0.001) (Fig. 2a).

Previous research has suggested that the colour of honey may influence the detection of H_2_O_2_, with darker honeys appearing to impair H_2_O_2_ recovery [20]. In the current study there was a slight negative trend between the Pfund colour of honeys and the maximum H_2_O_2_ detected by the assay, but this was not significant (*r*=−0.29, *R* ^2^=0.08, *P*=0.28) (Fig. 2b). This negative trend was improved when restricted to the jarrah samples, but did not attain significance (*r*=−0.78, *R* ^2^=0.60, *P*=0.07) (Fig. 2b).

Honey colour may also influence activity, as pigmented, antioxidant compounds such as phenolics and Maillard reaction products may either enhance or inhibit the antimicrobial activity of H_2_O_2_ [24]. Across the collection of honey samples there was no correlation between Pfund honey colour and antimicrobial activity (*r*=−0.07, *R* ^2^=0.01, *P*=0.79) (Fig. 2c), and no significant correlation when the analysis was restricted to jarrah honeys (*r*=−0.68, *R* ^2^=0.46, *P*=0.14) (Fig. 2f).

### Rapid degradation of H_2_O_2_ occurs in some honeys during the HRP assay

**Fig. 3.**
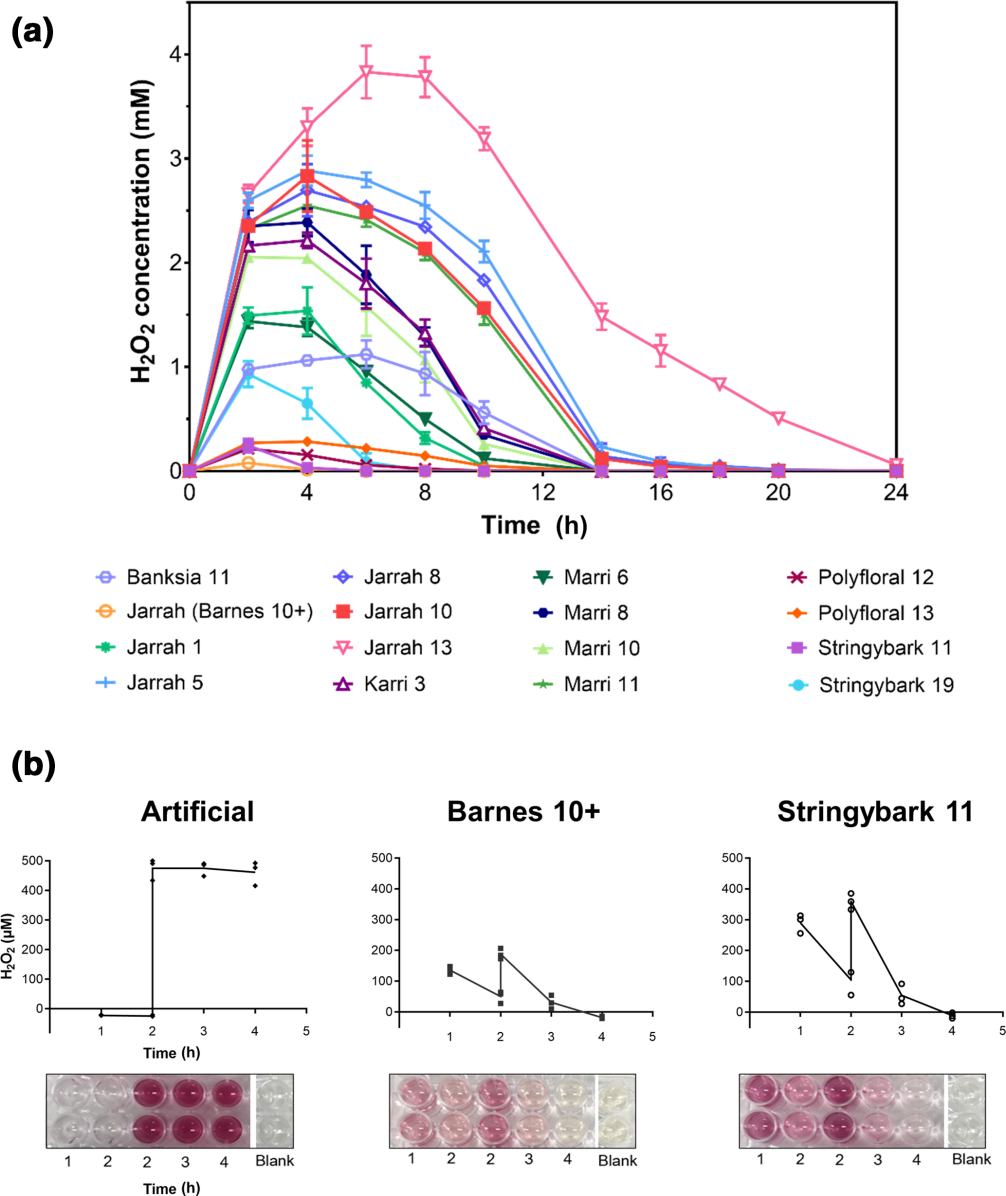
Rapid degradation of H_2_O_2_ occurred in some honeys. (**a**) H_2_O_2_ in honey samples detected by the HRP assay over 24 h; *n*=2 biological replicates (each with three technical replicates). The graph shows the mean and error bars represent the standard error of the mean (sem). There was substantial variation in the level and kinetics of H_2_O_2_ over the time course. (**b**) Barnes 10+ and stringybark 11 honey spiked at the 2 h timepoint with H_2_O_2_ (final concentration of 500 µM) showed rapid degradation of the H_2_O_2_ that was not seen in the artificial honey control. Representative images show colour changes seen in wells at each timepoint. *n*=3.

Plant-derived catalase and antioxidants have been suggested to quench H_2_O_2_ production in honeys [13, 25] and could be responsible for the variability seen between H_2_O_2_ detection by the HRP assay and activity measured by the bioassay. To test this, the kinetics of H_2_O_2_ production over time was compared across the different honey samples (Fig. 3a). The level of H_2_O_2_ produced over time differed widely in the different samples, and in the stringybark and polyfloral honeys this was extremely low, despite these samples having appreciable levels of antimicrobial activity as measured by the bioassay (Table 1).

To test whether H_2_O_2_ production was being degraded by a component present in some honey samples, artificial honey, Barnes 10+ and stringybark 11 honey samples were spiked with H_2_O_2_ and recovery was measured using the HRP assay. In the artificial honey sample, the level of H_2_O_2_ remained constant at 2 h post-spiking. In contrast, in the Barnes 10+ and stringybark 11 samples there was rapid H_2_O_2_ degradation with complete loss by 2 h (Fig. 3b).

## Discussion

Australian native flora have generated some highly active hydrogen peroxide honeys, particularly from flowering marri and jarrah trees of Western Australia [8]. A better understanding of the antimicrobial properties of H_2_O_2_ honey may enable expansion of the Australian honey market into financially lucrative clinical therapies that are currently enjoyed by manuka honey. However, this is currently impeded by high variability in antimicrobial activity among samples, including those derived from the same floral source [8–10] and difficulties in accurately assessing H_2_O_2_ production [20]. Here we show that peroxide-based active honey can retain useful activity over time and that there is an overall correlation of H_2_O_2_ production with honey activity, but there is also substantial variation among honeys. There is also direct degradation of H_2_O_2_ within some honey samples.

Almost all of the Australian peroxide honey samples retained a level of antibacterial activity considered to be potentially therapeutically useful (≥10 % phenol equivalence) after more than 10 years of storage, suggesting a useful shelf life under appropriate storage [16]. The mean decline in activity was 30 % over 11–12 years (Table 1), although there was substantial variation across honey samples, even though temperature and other conditions were identical. Marri honey samples exhibited a relatively low level of decline in activity that ranged from 11–29 %, while the stringybark samples lost 52–54 % of their activity (Table 1). In the 2011 Australian honey survey that collected the samples tested here, a similar set of peroxide honeys stored under identical conditions for 8–22 months had a mean decline in activity of 23 % [8]. This suggests there may be an initial rapid decline in antimicrobial activity that stabilizes over time. This is consistent with a study that measured the activity of honey samples every 3–6 months for 3 years and found the greatest decline in antibacterial activity occurred in the first 3–6 months [24].

Instability of glucose oxidase is thought to be the main reason for this decline in activity. Glucose oxidase is vulnerable to both light and temperature [7, 10, 11], but as all honeys were stored under identical conditions at 4 °C and protected from light this does not explain the variable rates of decline among them. Chemical changes in honey, such as the production of melanoidins from the Maillard reaction as honeys age, may also affect the stability and function of glucose oxidase, and these have been found to vary among honey samples [24]. Our data indicate that with appropriate storage peroxide-based activity is relatively stable, and this reduction in activity over time should not prevent honey from being a useful therapeutic, as all modern medicines have a shelf life. Understanding the divergent rates of decline between samples may help to standardize shelf life and promote peroxide honey in a therapeutic context.

In contrast to the stability during storage, we found rapid degradation of endogenous and spiked H_2_O_2_ occurred in some samples during the HRP assay, suggesting that a component present in honey was degrading H_2_O_2_ (Fig. 3b). However, despite this rapid loss of H_2_O_2_, these honeys had antibacterial activity of around ~10 % (w/v) phenol equivalence (Table 1). H_2_O_2_ degradation is thought to be caused by catalase introduced from pollen collected by bees [13, 26, 27], and some studies have reported that catalase is inefficient at destroying the low levels of H_2_O_2_ produced in honey, potentially explaining why antimicrobial activity remains [7, 28, 29]. Other antioxidant components present in honey might also be responsible for H_2_O_2_ loss by scavenging H_2_O_2_ and preventing detection by the HRP assay [3, 30], and these antioxidants may also have antimicrobial properties [3]. Future studies using fractionation may allow the specific components in honey to be identified and their interaction with H_2_O_2_ to be characterized.

A useful screening protocol for the antimicrobial activity of peroxide honeys would have a strong linear relationship between activity and the production of H_2_O_2_, which has been demonstrated between activity and MGO levels in manuka honey (*R* ^2^=0.95) [31]. To date, studies investigating the correlation between H_2_O_2_ and activity have been inconsistent. Some studies using diverse floral honeys reported a significant positive correlation [28, 32, 33], while others found no correlation for monofloral honeys such as honeydew [25], rapeseed or linden [33], or when a different bacterial species was tested [28]. In the current study a significant positive correlation was observed across all honey samples (Fig. 2a), but this was only able to explain the variation in activity in less than half of the samples (*R* ^2^=0.47). Low explanatory power is also apparent in the other studies where a correlation was reported [33, 34], and this may limit the usefulness of chemical assays based on H_2_O_2_ detection for honey screening. In our study, when the analysis was confined to jarrah honey the correlation improved and 95 % of the variation in activity was explained by variation in H_2_O_2_ production (Fig. 2a). Unfortunately, the small sample sizes meant we could not test this on the other floral honey groups and we do not know if this linear relationship is unique to jarrah honeys. Future testing of different monofloral honeys will determine whether useful linear correlations are possible for other honey types.

The poor linear relationship observed in this study between H_2_O_2_ production and activity may be explained by the H_2_O_2_ degradation noted above, by limitations in the HRP chemical assay and/or by complex interactions between different honey components. Previous studies have demonstrated that the level of H_2_O_2_ in honey that is detected by the HRP assay is significantly lower than the inhibitory concentration of H_2_O_2_ alone, suggesting that the assay is either not able to detect all H_2_O_2_ that is present or that the killing power of H_2_O_2_ is augmented by synergizing components in honey [34]. Pigmented Maillard reaction products have been implicated in suppressing H_2_O_2_ detection by the HRP assay [20] and darker honeys have been reported to produce less H_2_O_2_ [33], but we found no significant correlation between honey colour and our H_2_O_2_ measurements (Fig. 2c). It therefore seems more likely that there are complex interactions occurring in honey that can both degrade and synergize with H_2_O_2_.

There remain a number of challenges to using H_2_O_2_-type honey therapeutically. In addition to the difficulties in producing a reliable assay noted above, there remains a paucity of clinical or animal data demonstrating how honey works in infected wounds and skin. These can produce catalase that might directly degrade H_2_O_2_ [35], and they can contain multiple micro-organisms organized into biofilms that may respond differently to the simple bioassay used here, which is based on *S. aureus* alone. Future work aimed at understanding the complexity of honey and applying it in a real wound context will be very helpful for developing and marketing novel therapeutic honey products.

## Concluding comments

The relatively high level and the longevity of antibacterial activity in many of the Australian honeys tested here are promising for the future expansion of Australian honey into the growing global medicinal honey market. While the limitations of the HRP assay make it unsuitable for screening diverse honeys, it may be applicable to some monofloral honeys and it remains useful for determining changes in H_2_O_2_ production over time in single honey samples; for example, to study storage conditions and optimize shelf life. Further research on the specific interactions between H_2_O_2_, Maillard reaction products, plant-derived compounds such as catalase, phenolics and flavonoids, and potentially other honey components is needed to elucidate the antimicrobial mechanisms of honey and their potential interactions and create a more standardized therapeutic.
